# Extracts and Gleanings

**Published:** 1876-04

**Authors:** 


### Extracts and Gleanings.

*Atropia in Phthisical Sweating*—(*British Medical Journal*, *Nov.* 29, 1873.)—A notice in the *Philadelphia Medical Times* of last year, in which Dr. J. C. Wilson stated that he had successfully used sulphate of atropia, in doses of one-eightieth of a grain, for the relief of profuse sweating in four cases of phthisis, led Dr. Frantzel to make an extended series of researches in the Charite Hospital, in Berlin, on the effect of atropia in such cases. In a paper on the subject, published in *Virchow's Archiv.*, vol. lxviii., part i. (*Allgemeine Medicin. Central-Zeitung*, August 2), he states that, having given it to seventy-five cases, he has arrived at the conclusion that it is a remedy that he can confidently recommend, not only in phthisical sweating, but also in that which attends other diseased conditions, such as acute articular rheumatism and convalescence from trichinosis. Among the seventy-five cases were fifteen cases of more or less recent cheesy pneumonia, of whom all had more or less fever, with night-sweats; forty-eight of distinct pulmonary phthisis, of whom forty-two had hectic; one of acute articular rheumatism, with high fever; two of ulcerative endocarditis, and two of trichinosis. In the first fifteen patients, the sweating was in six completely arrested; in seven, much diminished; in two there was no change. In the forty-eight phthisical cases, the medicine had no effect in five, in twenty-one the sweating was remarkably abated, and in twenty-two it disappeared entirely. Several of the patients in whom the atropia failed were near death when it was given. In the eight cases of rheumatism, the atropia gave permanent relief in five; in two, it produced a marked diminution of the sweats; in one, it was useless. In one of the cases of ulcerative endocarditis it proved useful; not so in the other. In the two cases of trichinosis, the cessation of the acute stage of the disease, and of the hectic fever attending it, was followed without any rise of temperature by profuse night-sweats. Sulphate of atropia, in doses of a milligramme (.015 grain), was given two hours before the expected access of sweating, daily for five days in succession, in one case, and for three days in the other; the result being that the sweats entirely disappeared from the first evening when it was given. In one of the cases of rheumatism, in a man aged thirty-two, nearly all the large joints of the upper and lower limbs had been severely affected during five days; the patient was covered with sudamina, and, when seen by Dr. Frantzel, was bathed in sweat. A milligramme of sulphate of atropia was given immediately, and very soon there was an abatement of the sweating, which in two hours disappeared. It returned in the night, but ceased the next forenoon after the administration of a similar dose. The atropia was thenceforth given regularly night and morning, with the effect of completely preventing the sweating. The fever lasted fourteen days. In another case of acute articular rheumatism, atropia was given first in doses of one, then of two milligrammes, with a similar result; and it is remarked that on two days in the course of the disease in which it was omitted, the sweating recurred. The atropia was given according to the following formula: Sulphate of atropia, six milligrammes (9-100ths of a grain); extract of gentian, sufficient to make ten pills. Dr. Frantzel has never given larger doses than 1.2 milligrammes (a little less than one-fiftieth of a grain), from fear of producing toxic symptoms. Even doses of 0.6 and 1.2 milligrammes, though unattended with any mischief, have produced slight symptoms of poisoning. In not a few cases after taking the medicine, the patient felt itching in the neck, which, however, disappeared in one or two hours. The pupils not unfrequently acted slowly, and were sometimes dilated. In some cases there were muscæ volitantes. The atropia had to be stopped in four cases on account of diarrhœa; that this was due to the medicine was proved by the fact that it ceased when the atropia was discontinued, and reappeared when it was resumed. What the physiological action of atropia is in arresting perspiration, Dr. Frantzel says it is difficult to determine. He is, however, inclined to believe that the profuse sweats arise from relaxation of the walls of the vessels supplied to the sudoriferous glands; and he remarks that the researches of Meuriot, Fleming, Jones, Hayden and Brown-Sequard have shown that atropia contracts the smallest vessels. To this are to be ascribed both the diminution of the sweats, and the dryness of the mouth and skin, observed in cases of poisoning with belladonna and with atropia. —*Medical Times.*

*Diagnosis of Iritis.*—E. L. Holmes, of Chicago, in the *Chicago Medical Journal*, says: It seems to me our best works scarcely dwell with sufficient emphasis on the difficulties which may embarrass the student in the diagnosis of this disease.

A typical case of iritis, even in its early stages, offers little difficulty. The symptoms upon which a diagnosis is based are irregularity, contraction, and immobility of the pupil, discoloration of the iris, with loss of brillancy of its surface, turbid condition of the aqueous humor, pink zone around the cornea, photophobia in and about the eye, pain, and diminution of sight from turbidity of the aqueous humor, or exudation upon the crystalline lens.

Not unfrequently these symptoms are masked. From the very onset there may be not only considerable œdema of the conjunctiva of the globe, but also of the whole lid, with no marked change in the appearance of the iris or pupil. Such an attack could be easily mistaken for simple conjuctivitis. The terrible pain, so often experienced in iritis, is sometimes absent. The pupil is not always, at the commencement, irregular. In some forms of the disease the inflammation is confined almost entirely to the posterior layers of the iris, producing no exudation upon the pupillary edge, and scarcely any redness or irregularity.

Many of the symptoms of iritis are present in some forms of keratitis. We have redness, photophobia, contracted and immovable pupil, dimness of vision, and apparent turbidity of the aqueous humor, with apparent want of brilliancy of the surface of the iris from a cloudy condition of the cornea.

I allude to this subject, from the fact that vision of very many eyes is partially, or even totally, lost, because an improper diagnasis is made, and, in consequence, injurious treatment instituted. It is seldom that a mistake would be made, if the practitioner would take the trouble, in cases where doubt is possible, to instil a drop or two of solution of atropiæ sulph. (gr. iv to the ounce) in the eye. If iritis is present there is scarcely a possibility that the pupil will not dilate irregularly, which is, perhaps, the most important symptom in diagnosticating iritis.

I do not wish to dwell on the treatment of iritis, but will simply add that the free local use of atropiæ sulph. is of chief importance. The patient should be kept at rest, free from exposure to light. Pain may be relieved by subcutaneous injections of morphine. There is reason to believe that mild but continued doses of calomel serve, in a great degree, to shorten the course of the disease, although it is far inferior in value to atropine.

*Indications for Counter-Irritation.*—Dr. A. H. Smith expresses his opinion, in the New York *Medical Journal*, that there are four conditions in which counter-irritation may act to relieve pain or abate inflammation:

1. When there is a direct vascular connection between the irritated surface and the diseased part. In this case, counter-irritation acts by opening a larger passage for the blood through the sound tissue, and thus diverting the circulation in part from that which is diseased. An example of this is afforded by the application of blisters to the neighborhood of inflamed joints.

2. When a large surface is exposed to the irritating agent, in which case the entire circulation of the body, including that of the diseased part, may be modified. An example of this may be found in the use of hot foot-baths, to relieve congestions of the thoracic or abdominal organs, or of hot applications to the chest and abdomen to relieve wakefulness.

3. When the irritation is transferred to a distance by reflex action, and there simulates the first condition. As an example of this, may be cited the employment of blisters to the breast, to act upon the lungs, there being no direct communication between the vessels of the skin and those of the diseased organs.

4. When pain is the result of a mechanical cause, which cause may be removed by exciting muscular action through the reflex function; as when, in flatulent colic, the muscular coat of the intestines is brought into action by irritation applied to the abdomen, and expulsion of the flatus results. Of course, two or more of these conditions may co-exist, and probably in most cases such is actually the fact, and the result is thus rendered more or less complex.

*The Lungs—Breathing.*—All that live, down even to vegetables and trees, breathe—*must* breathe in order to live—live in proportion as breathe—begin life's function with breathing, and end its last in their last breath. And breathing is the *most important* function of life from first to last, because the grand stimulator and sustainer of all. Would you get and keep warm when cold, breathe copiously, for this renews that carbonic consumption all through the system which creates all animal warmth. Would you cool off and keep cool in hot weather, deep, copious breathing will burst open all those myriads of pores, each of which, by converting the water in the system into insensible perspiration, casts out heat, and refreshes mind and body. Would you labor long and hard, with intellect or muscle, without exhaustion or injury, breathe abundantly; for breath is the great reinvigorator of life and all its functions. Would you keep well, breath is your great preventive of fevers, of consumption, of "all the ills that flesh is heir to."

*Treatment of Uterine Tetanus.*—Dr. Alexander Milne, of Edinburg, after referring to the extreme difficulty of performing podalic version, where the liquor amnii is absent, or has long escaped, where the parts have become swollen and dry, and the uterus has contracted firmly around the fœtus, describes, in the *Lancet*, the means which he has found most beneficial in enabling version to be effected. In the first place, he considers chloroform should be administered, and very freely too; in many instances it relaxes the spasm in a wonderful manner, but he has seen it fail frequently in uterine tetanus. In the next place, when chloroform is ineffectual, recourse should be had to tartarized antimony and lobelia inflata. A grain of the former may be given, and twenty-five minims of the tincture of lobeblia inflata; after the lapse of twenty minutes the patient should be placed under the influence of chloroform. The triad form a potent antispasmodic whose conjoint action is almost irresistible, and the spasm is overcome. The uterus being relaxed, the hand finds entrance, and version becomes a possible thing. In regard to the hand to be employed, which he considers to be a matter of capital importance, he holds that the left, in the great majority of cases, is the proper one to employ. It is best in all dorso-anterior cases, and better success is attained with it than with the right, even in dorso-posterior ones, where there is fluid and space. Lastly, in reference to the point whether one leg or both should be seized, Dr. Milne observes that "in difficult cases, to speak paradoxically, we can neither afford to seize both nor to want both; that is to say, there is little room to enable us to get hold of both, but it is better to have both if at all practicable. The fœtus will then revolve more readily, and come down more speedily." Dr. Milne records a series of cases which support his views.

*Pruritus Pudendi in Pregnant Women.*—Dr. J. R. Block (*Cincinnati Lancet and Observer*) says: I think I am able to suggest a remedy for these cases, and which will reach the requirements of the practitioner better than any other hitherto employed. The manner of my coming upon it is the following: Some years ago a young man was, as he expressed himself, almost crazy with the intolerable itching attendant on an outbreak of urticaria. I had prescribed various remedies for the relief of the stinging formication. Everything, in fact, that I had previously employed, or which my recollection of remedies by standard authors had recommended, and they had all proven insufficient. Thrown upon my original resourcs for something that would give him at least a little rest, I bethought me of the chloral hydrate; which, on theoretical grounds, and from its properties of producing local anesthesia, ought to give my patient the relief he so urgently sought. Accordingly, I directed the following to be applied to the itching surface with a sponge:

R. Chloral Hydrate..........................dr. iij.
Aqua......................................oz. iv.

A single application gave prompt relief, and he was able to sleep after a forced abstinence of two nights. The tendency to a slight recurrence was immediately subdued by the further application of the solution to the parts affected; the final result was all that the therapeutist could desire. I determined to bear in mind the singular efficiency of the chloral in this case, and to give it a further trial at the first opportunity. I had only a few weeks to wait; the wife of a merchant, Mrs. F., pregnant three months, had endured for a week or two the most tormenting itching of the vulva. From instinctive modesty she had been deterred from mentioning the matter to a physician; relying for hope of relief on her own judgments, and the suggestions of her female friends; but all without avail. Unable to longer endure it, she applied to me April 28th. I prescribed the chloral hydrate, three drachms to four fluid ounces of water; to be applied to the parts affected once every hour till better. The application produced severe smarting, which, however, soon subsided; while the relief to the pruritus was prompt, decided, and almost permanent. Whenever the symptoms were again felt in the least, the same solution was applied; and after a few days the cure was complete.

*Treatment of Constipation aud Diarrhœa with Water and Tincture of Camphor.*—Every one knows how difficult defecation sometimes is for those persons who are afflicted with habitual constipation. In such cases relief is often sought from injections of cold water, with the occasional addition of oil or soap. The following clyster is recommended as infallible in such cases:

Take a tumbler and fill it half-full of water at the temperature of the room, pour in a few drops of tincture of camphor, just enough to give the water a slight sapidity, then fill the glass with water. Inject this slowly into the rectum till about sixty or eighty grammes have been introduced. At first no effect is perceived, but in about ten minutes the desire to defecate becomes irresistible. The effect becomes energetic in proportion to the quantity of tincture of camphor added. After the defecation it is well to repeat the injection of a small quantity of the same mixture and retain it in the rectum, which can readily be done, so as to prevent constipation on the following day.

Although at first sight it may seem rather improbable, the same remedy is also useful in checking diarrhœa, even though it may have been persistent. The injection should be extremely slow; after the dejection it should be repeated two or three times. The quantity of tincture of camphor should also be increased.—*Gazz. Med. di Roma.*

*Treatment of Intestinal Obstruction.*—In the *Gaz. Hebdomad,* (December 31, 1875,) two cases of successful treatment of intestinal obstruction are quoted. The patient in one case had had no dejections for nine days; there had been fecal vomiting for several days. The abdomen was tympanitic and distended. Injections had been tried thoroughly for several days without effect. Immediately afterward an injection of bicarbonate of soda (30 grammes) and tartaric acid (14 grammes) was employed. Carbonic acid being thus generated, a copious stool followed. This was repeated, and in a few days the patient was entirely well. In a second case where purgatives and clysters were also found ineffective, and the patient lay in a dangerous condition, with distended abdomen, prostration, and feeble pulse, pain, and vomiting, an injection containing Seidlitz powders (six packets of each, blue and white,) was entirely successful, and in a few days the patient was able to go about his business.

*Bromide of Potassa in Infautile Convulsions.*—By C. O. WELLER, M.D.—December 25th, 1873, Tom Dodson, a negro boy four and a half years old, was brought to my office to be treated for "fits." His parents informed me he had been having them almost daily for two or three months, and, though medical advice had been received and followed, he grew gradually worse. At this time the disease had made such progress as almost to have deprived him of the power of locomotion and of speech, and from a boy of ordinary intelligence he had lapsed into a state of almost idiocy.

From the history his parents gave me of his case—his symptoms during the attacks of convulsions—I concluded the trouble to be epilepsy. I gave him bromide potassium in liberal doses, and directed that he be brought to my office every week, that I might observe the effect of treatment.

After using the bromide about one month, he had very much improved, was more intelligent, could talk better, was regaining the use of his legs, and the convulsions were neither so frequent or severe. I ordered the same medicine continued. Six months from commencement of treatment, he had completely regained the faculty of speech, also his intelligence, and could run and exercise himself as other healthy children. He continues in that condition to this time—nearly two years from commencement of treatment—except that he will occasionally have a convulsion when from under the influence of the bromide; but whenever it is commenced again, and his system brought under its influence, the convulsions cease.

I report this case merely to add my testimony to that of others of the controlling influence of bromide of potassium over epileptic convulsions; and though we may not have in it a positively *curative* agent, yet one eminently *palliative.—N. O. Medical Journal.*

*Case of Rheumatism with Treatment.*—Dr. L. J. Davidson (*Cin. Lancet and Observer*) writes: Called March 14th, 1875, to see Mr. E. S., about twenty-two years of age; married the previous fall, had articular rheumatism of left wrist and right ankle, with tenderness on pressure of lumbar region, and cervicals of the neck; with co-existing and endocarditis and great febrile movement; confining him to his bed in an immovable position, a position he had been compelled to occupy many times previous. He being plethoric and of a congenital rheumatic diathesis. I apprehended hypertrophy of the heart. I at once prescribed full alkaline treatment; followed by quinia, frictions, etc.; omitting "firm dressings" and "hot packs." March 17. Able to walk upright across his room. March 20. Discharged, able to attend out-door business. After which time I made a visit of four days to see a friend who had written for me. On my return, March 31st, when stopped by Mrs. S., I found Mr. S. in about the same condition as before; the second attack coming on March 28th, with more cardiac pain. March 31st, 2 o'clock, P. M., I dispensed the following:

R. Podophylin....................................gr. ij.

Ol Sassafras.................................. m. x.

M.

*S.* To be repeated every four hours in treacle; alternately with

R. Fl. Ext., Valerian (Hance Bros.)......fl dr. ij.
Bicarb. Sodæ.............................. dr. ij.

*M. S.* To be repeated every four hours. Three of each were left for the night without any other palliative.

April 1st, 8 o'clock, A. M., being twelve hours later, I found all cardiac pain removed, and articulation free in every joint. Almost ten months have intervened without the least symptom of a recurrent attack. I have almost adandoned the use of opiates since I have been using Hance Bros.' and White's Fl. Ext. Valerian.

*Extensive Burns.*—Dr. P. V. Dorland (*Canada Lancet*) writes: During the intense smarting pain we are admonished to act promptly with the most efficient means at our disposal, for if this excitement be too long continued we may have complete collapse, or if not so serious an end, we may have, during this stage, congestion of some of the vital organs, and, at a later stage, perforation of the bowel from ulceration. By cutting short the pain we lessen the duration of the first stage, and the evil consequences of weakened nervous force and diminished circulation. By acting properly at this stage, we lessen the danger at the second stage, or that of re-action and inflammation. How often have we seen cerebral symptoms arising during the first stage, and, no doubt, at this stage also, begins those causes which result in perforation, a very common occurrence in the later stages. To relieve the smarting we simply require cold water and tr. cantharides; ʒj. of the latter to a gallon of water. This will relieve it in a few minutes, or cold water will do it alone, but requires a much longer time.

*Medicated Ice in Scarlatina.*—In a short communication to the *Lancet* (January 8, 1876), Mr. Edward Martin says:

"Every practitioner has at times to face the difficulties of the scarlatinal throat in young children. It may sadly want topical medication; but how is he to apply it? Young children cannot gargle, and to attempt the brush or the spray often fills them with terror. In many cases, neither sternness nor coaxing avails. If the doctor thinks it his duty at all hazards not to leave the throat untouched, the child is subjected to a struggle and a fright which probably render the proceeding more productive of harm than good. If, on the other and more wise side, he, when persuasion fails, goes no further, he is haunted by the feeling of not having done all that might have been done for the case. Most of us at times have been impaled on the horns of this dilemma. Yet, these little ones in almost every case will greedily suck bits of ice, as I doubt not most of your readers can testify. This has long been my chief resource, where I could not persuade the child to submit to the sulphurous acid spray. Lately I have been trying an ice formed of a frozen solution of the acid (or some other antiseptic), and I think my professional brethren will find it a valuable addition to their means. Though, of course, not so tasteless as pure ice, the flavor is so much lessened by the low temperature, and probably also through the parched tongue very little appreciating any flavor whatever, that I find scarcely any complaint on that score from the little sufferers; they generally take to it very readily. The process of making it is so simple that a few directions to any intelligent nurse will quite suffice; or, in urban practice, the chemist who dispenses the other prescriptions will undertake this one also. A large test-tube, immersed in a mixture of pounded ice and salt, is the only apparatus required, and in this the solution is easily frozen. When quite solid, a momentary dip of the tube in hot water enables one to turn out the cylinder of ice as the cook turns out her mold of jelly. I have tried the three following formulæ, all of which answer, though I think I prefer the first:

"1. Sulphurous acid, half a drachm; water, seven drachms and a half; mix and freeze.

"Chlorate of potass., one scruple; water, one ounce; dissolve and freeze.

"3. Solution of chlorinated soda, half a drachm; water, one ounce; mix and freeze.

"However, the form is of secondary importance, and each practitioner can construct his own. Boracic acid, salicylic acid, or any other harmless antiseptic with not too much taste, would, doubtless, be as useful as those I have indicated. It is the idea of applying them in the shape of 'medicated ice' that I recommend to the profession, with the belief that it is of practical value."—*Monthly Abstract of Medical Science.*

*On the Employment of Iodoform in Vaginismus.*—In the *Jour. de Med. et de Chirurgie Pratiques*, M. Tarnier relates a case of much interest, where iodoform succecded perfectly in relieving vaginismus.

A woman aged thirty-two, married seventeen years, was affected with an extreme hyperæsthesia of the vulva, producing pain on walking, coitus being extreme torture. There was no lesion of the vulva or neck of the uterus. The vulval aperture and the labia minora were dusted over with iodoform powdered, and a few hours afterwards the parts were insensible. Coitus was effected with scarcely any pain. A tampon of cotton-wool covered with the powder was placed between the lips of the vulva, and succeeded admirably.

M. Tarnier also employed iodoform in most intolerable fissure of the anus, which had resisted all the narcotics and astringents usually employed in such cases. After a single application the pain diminished considerably, and a cure was effected in a few days.—*London Med. Record.*

*Uterine Hemorrhage.*—In regard to the uterine disturbance that is frequently noticed at the change of life, Dr. Barker said that many of this class of cases came under his observation. The most common symptoms observable are an increased flow of blood, together with a slightly enlarged uterus. The patients were liable to be very much alarmed, and in dread of some possible danger. The treatment that was suggested was the use of rectal suppositories, one of them to be introduced three times a day. They were to be employed about a week before the epoch, and continued till menstruation appeared. The formula of the suppositories was as follows:

Ext. ergotæ aq............................... dr. ij.
Ol. theobromæ................................ dr. j.
Ft. suppos. No. xij.

The suppositories of ergot were preferred to hypodermic injections, from the unpleasant sequelæ of abscesses which were found occasionally to happen with the latter.

If the bleeding from the uterus continues, cones containing iodoform were introduced within the cavity of the uterus, and, in the experience of Dr. Barker, were of great benefit. The composition of these cones was:

Iodoform..................................... dr. ijss.
Gum Arabic.................................. gr. xv.

Mucilage sufficient to make ten cones.
—*N. Y. Medical Journal.*

*Diphtheria.*—Dr. Smith, in his work, says: As soon as the case comes under observation, the following mixture is applied every second or third hour over the fauces by one or two applications of a large camel hair pencil:

R. Acid Carbolic......................... gtt vi—x:
Liq. Ferri Subsulph.................. dr. iij.
Glycerine.................................. oz. j.
Misce.

If there is discharge from the nostrils, indicating diphtheritic inflammation of schneiderian membrane, a little of the same mixture, diluted with an equal quantity of warm water, is injected into each nostril every three to six hours. One-third to one-half a teaspoonful of the diluted mixture is a sufficient quantity to use for each nostril. This application, properly made, prevents decomposition, removes the offensive odor, and, which is of the greatest importance, prevents blood-poisoning.

Quinine, in doses of one or two grains, according to the age and severity of the case, is administered about every fourth hour, and each hour in the interval half a teaspoonful to one teaspoonful of the following:

R. Potass. Chlorat.........................dr. i ij
Tinc. Ferri. Chlor......... ...........dr. j
Syr. Simp..................................oz. iv M.

No drinks are allowed for a few minutes after its administration, as well as after the use of the brush, so as not to wash it away too quickly after the fauces.

*Treatment of Lead Colic in the Paris Hospitals.*—On the first day a purgative enema composed of infusion of senna, sulphate of 'soda, and powder of jalap is administered. This is followed by a purgative mixture called "eau de casse" (cassia), and is composed as follows: Decoction of tamarind 60 grammes, water 100 grammes, tartar emetic fifteen centigrammes—which the patient is to swallow in the course of the day; besides which he is to have a jug of decoction of guaiacum. In the evening an enema of walnut oil 125 grammes, ordinary claret 300 grammes. This is followed by a "bol calmant," composed of theriaca 4 grammes, opium 5 centigrammes. On the second day the patient is supplied with a drink composed of tartar emetic 25 centigrammes, water 500 grammes; this is facetiously designated "l'eau benite" (holy water). Besides this, he is treated with the same "tisane" (decoction of guaiacum), the same enema, and the same bolus, as on the first day. On the third day the patient drinks in the early morning two tumblers of a tisane, composed of equal parts of an infusion of senna and decoction of guaiacum, and in the course of the twenty-four hours, he is to take the same tisane, enema and bolus as on the preceding days. On the fourth day a purgative draught of senna and jalap, followed by the tisane of guaiacum during the day, and the bolus at bedtime. Fifth day: Two tumblers of laxative tisane ordered on third day, followed by the tisane of guaiacum, enema and bolus as before. Sixth day: Idem. Seventh day: The two tisanes, the same emena and bolus as previously. For the first three days the patient is condemned to *diete absolue*, which means total abstinence from food of any kind! This treatment, it must be confessed, must either kill or cure the patient, and strange to say, though purely empiricle, it is considered the classical treatment for lead colic in the Paris hospitals, and is said to have been instituted by the monks that were attached to the Charite Hospital many years ago. Fortunately for the patients, lead colic is, generally speaking, not a fatal disease.—*Medical News and Library.*

*Ammonia in Rheumatism.*—Dr. Franz Heeler (*Wiener Medizinische Presse*), speaks highly of caustic ammonia in rheumatism. For several years he had been a sufferer from muscular rheumatism; had taken all the common anti-rheumatic remedies, but with little alleviation, when he began to reason that in rheumatism, as in gout, there may be a uric acid diathesis; he thought that liquor ammoniæ, on account of its rapid volatilization, would be the remedy most readily absorbed, and the most prompt in action. He used the ammonia and was almost instantly relieved. The remedy, he claims, has proved a positive cure in all recent cases of muscular rheumatism which have fallen under his observation; he cites numerous cases in which relief, as instantaneous as his own, was experienced.

He also observed its effects in several cases of acute articular rheumatism, in two of which six drops sufficed to subdue the pain and swelling within a period of twenty-four hours.

In a case of chronic rheumatism of a finger joint, which had lasted for over half a year, the simple administration of the ammonia completely dispelled the inflammation and pain in the joint within two days.—*Clinic.*

*Chloral.*—The uncertainty of the action of chloral, however rare, is an undoubted fact which lessens very much the safety of the drug. Some time ago we expressed the opinion that unexplained death after chloral was more common than after any other of the narcotics in common use. Two cases reported in the daily papers since our last issue affords fresh support to this belief. In one case a medical assistant, on whom an inquest was held at University College Hospital, was in the habit of taking hydrate of chloral to induce sleep. He was found in the street dying, as if from the influence of some narcotic poison, and when brought to the hospital was actually dead. In the other case a lady, said to have been subject to epileptic fits, had a small dose of chloral (10 grams) administered to her, and was found the next morning lying on her face, and dead. Death after an epileptic fit from accidental suffocation is not uncommon, and it is very possible that it was in this way that it happened. But if a fit occurred during the night it does not appear to have been sufficient to rouse the husband of the lady who was sleeping by her. The habitual dose of chloral cannot be acquitted (in the absence of any direct evidence of a fit) of having contributed to the fatal result. Each case points the lesson very strongly that the nocturnal use of chloral is not without its dangers, and should as much as possible be discouraged.—*Lancet.*

*Treatment of Gonorrhœa.*—In the Cincinnati *Lancet and Observer,* Dr. Minor gives some comparative cases of the treatment of gonorrhœa. He concludes:

Other cases might be mentioned, but let the foregoing suffice, as in all the result was about the same. In every case the too long use of the injections used was carefully guarded against, and the patient received the usual directions regarding diet, etc., generally laid down in the text-books on this special subject. The reader will at once see that the duration of a case of gonorrhœa is quickly shortened when copaiba is used in place of oil of sandal. In case of gleet, on the contrary, the oil of sandal far excels the balsam of copaiba, and all injections may be omitted also while the sandal is being used.

The following conclusions in regard to the relative value of the two drugs in the treatment of gonorrhœa and blenorrhœa, are drawn from my experience with these agents:

1. In cases of accute gonorrhœa occuring in strong and healthy persons, without any tendency to kidney trouble or gastrict derangement, the use of balsam of copaiba will be found to be the most satisfactory, as compared to sandal-wood oil.

2. In cases of gonorrhœa which show a tendency to become chronic, and in which the continuous use of copaiba has caused irritation of the kidneys and a gastric derangement, sandal-wood oil may be substituted with great advantage.

3. In cases of obstinate gleet, oil of sandal-wood in large and long-continued doses will, without the aid of other means, completely cure the malady.

4. Sandal-wood oil is agreeable to the taste, and creates no nausea, nor does it communicate a disagreeable odor to the breath and urine, as in the case of balsam of copaiba.

*A Question on the Physiology of the Brain.*—The present generation of medical men have been taught that lesions of either hemisphere of the brain communicate their results to the opposite side of the body. The decussation of the fibres at the base of the brain furnished a ready explanation of the phenomenon. But now this must be unlearned. Brown Sequard has collected 150 cases of paralysis in which the brain-lesion was on the paralyzed side. He says: "The character of the symptoms in brain-diseases is not in the least dependent on the seat of the lesion, so that a lesion of the same part may produce a great variety of symptoms, while on the other hand the symptoms may be due to the most various causes—various not only as regards the kind, but also the seat of the organic alteration. In view of these facts," he continues, "I have been led to believe that lesion of the brain produce symptoms, not by destroying the function of the part where they exist, but by exerting over distant parts an inhibitory or an exciting influence, or in other words, either by stopping an activity or setting it in play." These views are in direct opposition to the doctrines of phrenology.—*Pacific Med. and Surg. Jour.*

*Treatment of Albuminuria During Pregnancy.*—Dr. Tarnier, chief physician of La Maternite de Paris, extols, in *Le Progres Medical*, the efficacy of a milk *regime* in cases of albuminuria, occurring in pregnant women, and regards it as a preventive treatment of eclampsia. Knowing the insidious manner in which albuminuri adevelops itself, he examines the urine of all women who present themselves, and those who are suffering from albuminuria he places on the milk diet, allowing nearly two pints of milk, and only two meals on the first day, and increasing the former and diminishing the latter until the fourth day, when the patient gets seven pints of milk and no other food or drink. In grave cases, however, he does not observe this gradation, but commences by giving the full quantity of milk. Under this treatment, Dr. T. says, after giving it a most extensive trial, the albumen rapidly decreases or disappears from the uterine in from eight to eleven days. He considers that in almost all cases of eclampsia the cause is to be found in modifications produced in the organism by pre-existing albuminuria, and that curing the latter is the surest means of preventing the former.—*The Doctor.*

*On the Treatment of Phagedenic Gangrenous Veneral Sores.*—By B. D. Simmons, M. D.—Within the last two years several cases which will admit of this classification have come into the hospital for treatment. The hot iron, nitric, chromic, and carbolic acids were all tried in turn, as well as "Ricord's born enemy to phagedenic," with what we believe to be the average rate of success.

The last four cases, and one especially, which we shall refer to, was almost entirely treated, however, by, if not a new process, one which was productive of such satisfactory results as to warrant us earnestly recommending its trial. This consists in the continuous immersion of the diseased part in hot or warm water.

. . . It is our opinion that the destructive agency is to be found in the peculiar or specific character of the discharge, and that the water simply removes or dilutes it, so as to destroy its action, the same as it would with a caustic.—*Medical Record.*

*Bromide of Lithium.*—M. Rouband draws the following conclusions from his investigations of this drug: Bromide of Lithium, as a medicine, has a double effect. It possesses, in a high degree, those lithotriptic properties attributed to the salts of lithia, and, in addition, like other bromides, affects reflex sensibility most energetically. It has not, however, the inconvenient action on the heart displayed by bromide of potassium. Consequently, its place in therapeutics is in the first rank among lithiasics and among sedatives, and its action is particularly valuable in the uric acid diathesis, which is accompanied by painful symptoms, and in neuroses, which are so often complicated by the presence of uric acid.—*Bull. Gen. de Therap.*

*Baths of Chloral Hydrate in Confluent Small-Pox.*—At the Society des Hospitaux, after the reading of the report upon prevalent diseases, M. Dujard. Beaumetz, at present in charge of the temporary female hospital, remarked that he had obtained the most beneficial effects from full baths of chloral hydrate, in cases of confluent small-pox; the baths were used at the period when the epidermis detaching itself *en masse*, leaves the dermis exposed.

The dose of the chloral for a bath never exceeded twenty grammes; the results were not alone a thorough disinfection of the patient, but also a prompt cicatrization of the skin.—*Bulletin General de Therapeut, November.*

*Hypodermic Injection of the Neutral Sulphate of Atropine in a Case of Reflex Contraction.*—At the Chirurgical Society (Paris) the Secretary-General read a report of three observations of M. Spillman. On the 11th July, 1875, this surgeon saw a child sixteen years of age in whom the movements of the elbow joint had become very difficult, on account of a fall on that part of the body; there was, however, neither fracture or luxation. It was treated with cataplams and compresses of lead water. The treatment, however, gave no results; and although there was neither redness nor tumefaction of the elbow joint, still the forearm remained flexed; the biceps and triceps were hard like cords, and all attempts to straighten the limb caused very severe pain. Explorations of the brachial plexus and of the vertebral column revealed no lesion. Having exhausted everything recommended in such cases, M. Spillman recollected that M. Dubrueil had reported to the society that in a similar case he had resorted to the hypodermic injection of morphia, and was about to adopt this when a case came to his knowledge in which the injection of the neutral sulphate of atropia had been followed by the most happy effect.

The injections were made and repeated through a period of time, and the patient was entirely cured. The dose injected varied from 1 to 3 milligrames, according to the toleration of the patient for the agent.—*Bulletin General de Therapeut, for November.*

*Treatment of Eczema in Children.*—Dr. Calpari, (*Bull. de Ther.*) extols the effect of lime water in curing eczema of the head and empetigo of the face in children, especially chronic diseases, which have resisted other treatment, and states that a marked improvement is noticeable after using it for eight days. He recommends it to be taken in quantities varying up to half a pint, according to the age of the patient, and to dust the part with carbonate of magnesia; but the latter he only considers necessary when the secretion is very irritant.

*Delirium Tremens — Treatment with Lupuline.*—Lupluline in large doses has proved efficacious in combating the insomnia of delirium tremens. One man, an athlete, had been unable to sleep for ten days, and it was decided to administer to him large doses of lupuline—three drachms were given in eight ounces of ale every two hours. During eight hours he had taken nine drachms of the drug, and at the end of that time he went to sleep. The usual dose is two drachms every two hours. The remedy has been had recourse to successfully in six cases.—*N. Y. Med. Jour.*

*Counteracting the Nausea of Opiates with Belladonna.*—Acting on the known advantage of uniting atropia with morphia in hypodermic injections, Dr. Green, the house-physician, tried the combination of belladonna with opiates in their administration. His method is to use rectal suppositories, each containing two grains of opium and two grains of the extract of belladonna. No nausea follows the use of opium when thus administered, and it has been used in a sufficient number of cases to prove its advantage.—*N. Y. Med. Jour.*

*Mistura Ferri Chloridi Composita.* (*Basham's Mixture.*)—

R. Liquoris ammonii acetatis........ f. oz. iii.
Tincturæ ferri chloridi............. f. dr. iiss.
Acidi acetici diluti.................. f. dr. i.
Curacoa vel alcohol................. f. dr. ii.
Syrupi...................................
Aquæ................................ *aa* q. s. ad f oz. vi.

Fiat mistura. Signa—Dose, a tablespoonful.

### SCIENTIEIC GLEANINGS.

BY R. C. WORD, M.D.

*Chemical Discoveries.*—The new and useful compounds, both in medicine and the arts, introduced by modern chemistry are truly wonderful. The once useless and ill-smelling *coal-tar* has, perhaps, yielded a greater and more useful variety of compounds than any substance known.

From coal-tar has been derived *benzole*, *toluole*, *xylole*, *cumole*, *cymole*, etc., etc. Benzole possesses great readiness for forming new compounds by uniting with other bodies. By varying its component atoms an almost infinite variety may be produced; with nitric acid it forms nitro-benzole—a poison resembling the oil of bitter-almonds. This substance, by distilling with iron-filings, and acetic acid produces *analine*. Analine was first obtained from indigo, but is also found in coal-tar. *Carbolic acid* is obtained from coal-tar; and from carbolic acid has been recently derived *salacylic acid*—a substance likely to prove exceedingly valuable as a medicine and as an antiseptic. *Picric acid* is likewise obtained from carbolic acid, and is useful as a yellow dye. With certain alkalies it forms compounds which are terribly explosive.

*Leather.*—A new fabric—*cuir-liege*—has been introduced into France as a substitute for leather. It consists of sheets of cork coated with rubber and a textile substance; said to be lighter, cheaper, and more durable than leather.

*Comparative Psychology of Man.*—Herbert Spencer, in *Popular Science Monthly*, says, upon this subject, that "throughout the animal kingdom the union of varieties that have become widely divergent is physically injurious; while the union of slightly-divergent varieties is physically beneficial.

"Some facts seem to show that mixture of human races extremely unlike, produces a worthless type of mind. To natures respectively adapted to slightly-unlike sets of social conditions may be expected, by their union, to produce a nature somewhat more plastic than either. The best genius of England resulted from a union of Celts and Anglo-Saxons."

*Conflict of Science and Religion.*—Touching the conflict between Religion and Science, Dr. LeConte, in a late article, remarks that, some centuries ago, great theological disgust was produced by the announcement that the sun, and not the earth, was the centre of the planetary system. Recently some annoyance has resulted from the finding of human remains in situations where they ought not to have been, according to present theories; and, yet more recently, great dissatisfaction is expressed at the possible derivation of man from some inferior organism. Yet all these facts, but the last, have been accepted after much bitter controversy, and the fountain of spiritual truth remains unclouded and undiminished.

*The Spectroscope.*—It has been ascertained, by spectroscopic observation, that the sun and all the visible heavenly bodies are composed of the same elements which are in the earth, with the exception of a supposed element in the sun, to which the name of "helium" has been given.

*Snow as a Fertilizer.* —The opinion so common among agriculturists, that heavy snows are generally followed by fine crops, finds confirmation in recent observations, which show that the dust gathered in the atmosphere and brought to the earth by the falling snow yields, on analysis, 53 to 61 per cent. of ash, and 47 per cent. of organic matter.

